# Association of Infant Eczema with Childhood and Adult Asthma: Analysis of Data from the 1958 Birth Cohort Study

**DOI:** 10.3390/ijerph15071415

**Published:** 2018-07-05

**Authors:** Ghada Abo-Zaid, Richard A. Sharpe, Lora E. Fleming, Michael Depledge, Nicholas J. Osborne

**Affiliations:** 1 European Centre for Environment and Human Health, University of Exeter Medical School, Knowledge Spa, Royal Cornwall Hospital, Truro, Cornwall TR1 3HD, UK; abozaidg@gmail.com (G.A.-Z.); rsharpe@cornwall.gov.uk (R.A.S.); l.e.fleming@exeter.ac.uk (L.E.F.); m.depledge@exeter.ac.uk (M.D.); 2 Department of Mathematics and Statistics, Ain Shams University, Khalifa El-Maamon St, Abbasiya Sq., Cairo 11566, Egypt; 3 Public Health, Cornwall Council, New County Hall, Truro, Cornwall, TR1 3AY, UK; 4 School of Public Health and Community Medicine, University of New South Wales, Kensington, Sydney 2052, Australia

**Keywords:** asthma, eczema, atopic march, longitudinal cohort study

## Abstract

The influence of early life exposures on later life disease has for some time provided clues to modifiable risk factors of disease. The “atopic march” is thought to play a role in the progression of allergic diseases and may offer an opportunity to lower asthma’s health and socioeconomic burden, although evidence remains controversial. We aimed to examine the relationship between early life eczema and asthma later in life. Using the National Child Development Study, we examined infant eczema and childhood and adult asthma. Data related to asthma or wheezing bronchitis were available for 13,503 (73%; 95% CI 72–74), 11,503 (61%; 95% CI 60–61), 12,524 (68%; 95% CI 67–69), 11,194 (60%; 95% CI 60–60), 9377 (51%; 95% CI 51–51), and 9760 (53%; 95% CI 52–53) subjects at ages 11, 16, 23, 33, 44, and 50 years, respectively. Logistic regression models were fitted to examine each wave separately before and after adjusting for a range of potential confounders. Generalised estimating equation (GEE) methods were undertaken to examine the associations after pooling all data from questionnaires. The prevalence of self-reported asthma in those that had previously reported infant eczema ranged from 1.0%; 95% CI 0.9–1.4 (age 44 years) to 2.2%; 95% CI 2.1–2.3 (age 33 years). Participants with infant eczema had a 2–3-fold increased risk of reporting asthma in childhood and adulthood; this was 1.6 times at age 44 years when using spirometry measures. Similar effect sizes were observed in the GEE models when considering all participants (OR 2.9; 95% CI 2.6–3.2). Childhood and adult asthma were consistently associated with infant eczema both by using the self-reported data and lung measures.

## 1. Introduction

The prevalence of allergic diseases represents a significant burden on societies and healthcare systems worldwide [1]. Researching potential causal pathways has led to hypotheses into the development of early life eczema and the progression to other allergic phenotypes such as asthma in later life [2]. Case definitions and changing measurements have been found to limit previous studies investigating allergic diseases [3]. A shift from subjective (e.g., self-reported) to objective (e.g., spirometry and total IgE/specific IgE) measures to define asthma will enable a different measure, and reduce the bias of memory decay.

Eczema often occurs in early childhood, and around 50% of patients later develop rhinitis or asthma [4,5]. Similarly, asthma is thought to affect around 20% of children and 10% of adults in the UK [6]; it represents a continuing public health concern because there appears to be no global downward trend [7].A range of genetic, behavioural, atopic sensitisation, and environmental risk factors (e.g., smoking, breastfeeding, socioeconomic status, and housing) influences the risk of eczema and asthma [8,9]. Furthering our understanding into the associations between parental behaviours, socioeconomic status (SES), and housing type may shed further light into these complex diseases.

The association between childhood atopic eczema and asthma has been reported in both cross-sectional and longitudinal study designs [10,11,12,13]. Prior research has shown that the risk of developing asthma after being diagnosed with eczema in childhood ranges from 25–80% [10,11]. More recently, research has detected the influence of early life either during “foetal origins” or during childhood on later life disease or condition [12]. However, this paradigm suggests that differences in a population’s environmental exposures (e.g., parental smoke status), especially in the in utero and perinatal periods, may provide evidence of a pathological pathway. While there is evidence to support this, models of higher complexity are needed to further our understanding into the aetiology of these complex heterogeneous diseases at the population level. This can be achieved through the utilisation of large secondary datasets and longitudinal modelling.

In this study we use the UK’s National Child Development Study (1958 Birth Cohort (1958BC)) to investigate associations between infant eczema and asthma in childhood and adulthood. We aimed to examine whether infant eczema perhaps associated with a progression of childhood asthma, and then whether it persists into adulthood. We also investigated whether infant eczema is still an independent factor after accounting for parental behaviour (e.g., parent’s smoking behaviour during subjects’ childhood, mother’s smoking behaviour during pregnancy, breastfeeding, mother’s weight at birth, low birth weight of child) and socioeconomic status (SES) (assessed via father’s occupation). Furthermore, we stratified the data based on range of factors such as sex and accommodation type (social housing or owner’s occupied) to examine whether these factors have an influence on the association between infant eczema and childhood/adulthood asthma.

## 2. Materials and Methods

### 2.1. Study Population and Data Collection

The 1958BC study is a longitudinal cohort study that was originally begun to assess the social and obstetric factors associated with still birth and death in early infancy, and has been previously described [14]. Briefly, participants were recruited during one week in 1958 that had over 17,500 births across the UK [13]. The cohort was retraced at age 7 years (1963) where questionnaires on the child’s health, education, and physical and social development were completed by a parent or guardian. Follow-up surveys were conducted at ages 11 years (1969) and 16 years (1974). The survey contained questions about asthma and wheezing at ages 11 and 16 years, as well as other information including parental occupation, parent’s accommodation, parental smoking status, and birth weight.

### 2.2. Follow-Up

The participants were interviewed during adulthood at ages 23 (1981), 33 (1991), 42 (2000), 46 (2004), 50 (2008) and 55 years (2013). Anthropometric, biochemical, and spirometry measurements were undertaken when participants were aged between 44 and 45 years (September 2002–December 2003). Data related to asthma or wheezing bronchitis were available for 13,503 (73%), 11,503 (61%), 12,524 (68%), 11,194 (60%), 9377 (51%), and 9760 (53%) subjects at ages 11, 16, 23, 33, 44, and 50 years, respectively. During childhood, the participants were followed through school, although almost 47% were lost to follow-up by 2013 [14].

### 2.3. Self-Reported Outcomes

**Figure 1:**
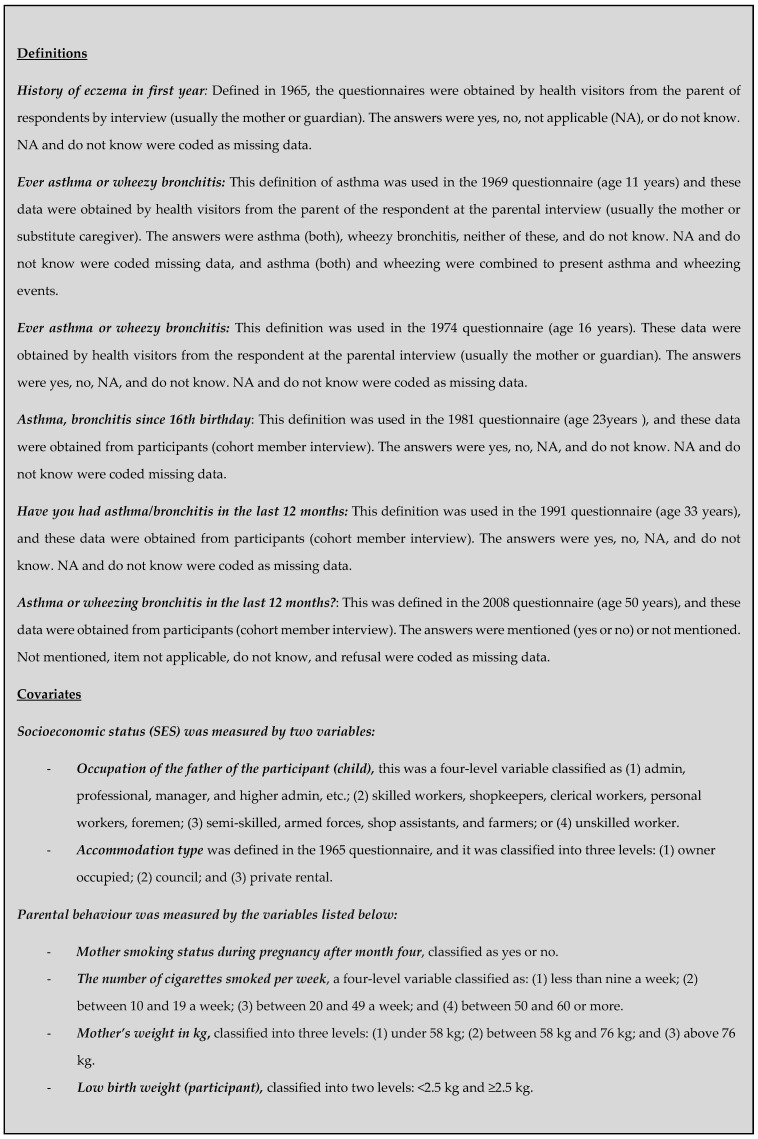
Brief explanation for the definitions and covariates that have been considered in the text.

Despite known limitations, self-reported outcomes for eczema and asthma are used as case definitions [15]. The survey collected information about a range of covariates and outcome definitions (Figure 1). A number of different questions were used to define an asthma case, which varied by wave (year of survey). Measures were examined to define asthma ever and current asthma. For example, at age 7 years, the question was “Has the child ever had (a) Attacks of asthma?”; at age 11 and 16 years, participants were asked “Has the child ever had attacks of: Asthma/Wheezy bronchitis?”; and at age 33 years the subject was asked if he/she had “ever been told (he/she) has asthma”.

For consistency, we excluded age 7 years because the question that was used to measure asthma differed to the other waves used in this study (i.e., did not include wheeze). Spirometry data wer taken at age 44–45 years, where we defined asthma as participants have an FEV1/FVC of <70% [16]. Unless otherwise stated, we refer to these outcome definitions as asthma throughout the paper (Figure 1). The interaction between the spirometry and total IgE was used to define the allergic asthma cases. The ROC nonparametric analysis was used to determine the cut-off point of total IgE (IgE > 168 k/UL). The analyses were repeated to examine the effect of infant eczema on asthma cases with cut-off points (FEV1/FVC of <70% and total IgE > 168 k/UL).

We pooled all questionnaire data to consider the repeated measurements of the event (asthma) for the same participant from childhood to adulthood. The analyses were repeated for all ages groups after excluding the participants who said they smoked tobacco. In addition, we repeated the analysis stratified by sex and accommodation type (homeowners versus council government-supplied housing).

### 2.4. Statistical Analysis

Univariate models and multivariable logistic regression models were constructed. Models were adjusted for parental behaviour and SES in a cross-sectional study for ages 11, 16, 23, 33, 44, and 50 years. Four logistic regression models were fitted for each wave separately, to examine whether there was an association between infant eczema and asthma, before and after adjusting for other covariates. Model 1 is an unadjusted logistic regression model where the association between infant eczema and asthma is examined. Model 2 adjusts for sex and SES (father’s occupation, accommodation at age 11 years). Model 3 extends this model by adjusting for parental smoking status and mother’s smoking status during the second trimester of her pregnancy. Model 4 includes the addition of child weight at birth and mother’s weight during pregnancy into the final adjusted model. However, the main focus will be on Model 1 (unadjusted model) and Model 4 as an adjusted model, while the details for the remaining model findings will be presented in the appendix. These analyses were completed for all of the included participants and then repeated after excluding smokers. The maximum likelihood method was used to estimate the parameters. All of the confounder factors mentioned above were selected based on previous studies [8,17,18,19].

In our longitudinal model, we used the generalised estimating equation (GEE), a repeated measurement model, to account for the correlation for the repeated measurement of the event (asthma) for the same participants from childhood to adulthood. The GEE was fitted for the same four models described above to account for the potential variation between waves and the correlation of repeated measurements of asthma for the same participant from childhood to adulthood (after pooling all of the data together). Where smokers were excluded from the sample, it reduced the number of participants in the analysis: 217 (16.45%), 579 (65.57%), 959 (46.85%), 492 (37.82%), and 273 (28.80%) participants reported smoking tobacco at age 16, 23, 33, 44, and 50, respectively. We selected an approach in building the four models, as opposed to a step-wise approach, as many of the potential confounding factors had been clearly illustrated in numerous asthma cohort studies internationally [8,17,18,19].

## 3. Results

### 3.1. Prevalence

**Figure 2:**
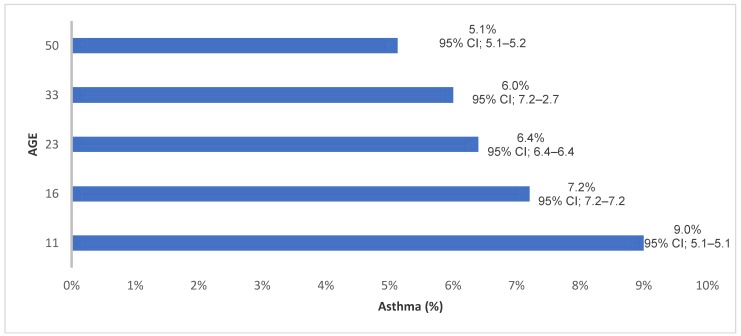
The prevalence of current asthma or wheezing/bronchitis in the 1958BC study from 11 to 50 years of age.

The self-reported prevalence of current asthma or wheezy bronchitis decreases with increasing age (Figure 2). The self-reported prevalence of asthma was highest in childhood at the ages of 11 years (9.0%) years, while it was the lowest at age 50 years with a prevalence of asthma at 5.1% of the population. Participant demographic and lifestyle characteristics and risk of asthma varied across the waves (Table 1). The highest prevalence of the participants who had eczema and self-reported asthma across the waves were at age 33 years (2.2%), while the lowest prevalence of asthma and eczema was 1.0% at age 44 years, when spirometry data were used to diagnose asthma (Table 1).

The proportion of asthma cases associated with the four occupation categories for the participant’s father remained approximately similar across the waves. Group II (i.e., skilled workers, shopkeepers, clerical workers, personal workers, foremen) had the highest proportion of asthma cases (ranged from 50% to 55% for all ages groups). Men aged 11–23 years and women aged 50 years had a higher prevalence of current asthma (Table 1).

### 3.2. Eczema and Risk of Asthma (Logistic Regression Model)

#### 3.2.1. Self-Reported and Spirometry Data

There was a consistent positive association between infant eczema and ever having asthma (self-reported data) in childhood and adulthood, which was slightly higher in Model 1, at age 11 years (OR 3.76; 95% CI 3.14–4.49) when compared to age 33 years (OR 2.33; 95% CI 1.92–2.79) and age 50 years (OR 2.55; 95% CI 2.00–3.25) (Table 2). The effect size was smaller at age 44 years in Model 1 when we used a different case definition (i.e., spirometry) in analyses for all data (OR 1.36; 95% CI 1.06–1.75) and when smokers were excluded (OR 1.69; 95% CI 1.27–2.25) (Table 3). However, the evidence was not consistent across Models 3 and 4 at age 44 years, where there was no association between eczema and asthma prior to excluding participants who smoked tobacco. The association remained after excluding tobacco smokers (Table 3).

#### 3.2.2. The Interaction between Spirometry and Total IgE (Atopic Asthma Cases)

We re-analysed the data by using the interaction between spirometry with cut-off point (FEV1/FVC of <70%) and total IgE (with cut-off = 168 k/UL). This yielded 189 cases with a prevalence of asthma of approximately 3% at ages 44–45 years. The effect size was slightly higher at age 44 years compared to spirometry and total IgE measures for all data (OR 3.46; 95% CI 2.19–5.17) and after excluding smokers (OR 5.17; 95% CI 3.10–8.10). The findings were still significant for adjusted Models 2, 3, and 4 (Table 3). However, the 95% confidence interval was slightly wider if compared to the results obtained from the spirometry measure, probably due to the lower numbers. The effect size of eczema was slightly higher after excluding smokers, which provides evidence that eczema is a risk factor for the adult allergic asthma endotype.

#### 3.2.3. Eczema and Longitudinal Risk of Asthma (the GEE Model)

We assessed the percentage of repeated asthma/wheezing or bronchitis cases (having self-reported asthma at more than one time point) from ages 11 to 50 years (Table 4). The highest percentages of persisting asthma/wheezing or ever having bronchitis were at ages 16 and 50 years, as 53% of asthma cases at age 11 years were still reported at age 16 years, and 56% of asthma/wheezing or bronchitis cases at age 33 years were still present at age 50 years. However, the highest percentage of persisting asthma at age 16 years (53%) may be an overestimation since the self-reported question at ages 11 and 16 years was “have you ever had asthma or wheezing bronchitis?”, indicating that there may be duplicated asthma cases at age 16 years. To overcome this problem, age 16 years was removed from the longitudinal analysis (pooled data were only for ages 11, 23, 33, and 50 years) to avoid overestimated findings.

Using the GEE model, there was a consistent 3-fold increased risk for the onset of asthma in participants with infant eczema (in all four adjusted models). The association held both before and after excluding participants who smoked at different ages across the same four models. Men had an almost doubled risk of asthma if they had infant eczema when compared to women, which was apparent both before (OR 4.0; 95% CI 3.5–4.4 versus OR 2.1; 95% CI 1.8–2.4) and after excluding participants that smoked during the study (OR 4.3; 95% CI 3.7–4.8 versus OR 2.2; 95% CI 2.0–2.5). The trend between males and females held across all of the models.

Accommodation type did not appear to be a modifiable risk factor (Table 5). While the confidence intervals overlapped, participants with infant eczema and living in owner occupied housing may have a slightly higher risk of asthma when compared to those residing in rented accommodation. In Model 1, participants with eczema and residing in owner occupied houses (OR 3.6; 95% CI 2.8–3.7) had a higher risk of asthma in adulthood when compared to renters (OR 2.6; 95% CI 2.1–3.1). The same findings were revealed in the fully adjusted models (Table 5).

## 4. Discussion

### 4.1. Main Findings

In this study, infant eczema was a significant risk factor throughout childhood and adulthood for asthma, even after adjusting for a range of covariates. The higher risk of self-reported asthma was greater during childhood across waves (Figure 1). We found that effect sizes were greater in analyses using self-reported health outcomes and the intersection between total IgE and spirometry when compared to the analyses at age 44 years, at which point we used participants’ spirometry results. In spirometry data, the effect size was not significant between infant eczema and asthma until stratified by tobacco smoking. The overlap between “asthma or wheezing” as a definition of asthma may have led to an overestimation of the findings when compared to the spirometry data. However, both measures (self-reported, spirometry, or the intersection between the total IgE and spirometry) yielded a significant association between early life eczema diagnosis and developing asthma. These results further highlight the increasing awareness and changing diagnostic habits required to make a precise evaluation of epidemiologic trends. This is especially true in long-running birth cohorts with difficulty in defining health outcomes, such as in the absence of a gold-standard test for asthma in the 1960s [20].

### 4.2. Comparison with Other Studies

Other authors have shown an association between childhood atopic eczema and asthma either by using cross-sectional or longitudinal designs. Thomsen et al. (2005) examined the association between asthma and eczema in adulthood among Danish twins aged between 12 and 41 years [21]. While the confidence intervals overlap, Thomsen et al. [21] also revealed that the risk of asthma in men (OR 3.45, 95% CI 2.22–5.37) may be higher when compared to women (OR 2.03, 95% CI 1.49–2.79) for participants aged between 12 and 41 years old. Similar to our findings, infant eczema was associated with adult asthma [22,23]. The authors relied on a self-reported case definition from a 1994 survey, and the results may also be influenced by a range of biases and overestimation of the association. To overcome some of the limitations of this study, we compared the results from logistic regression and GEE models. Burgess et al. also concluded that childhood eczema increased the likelihood of childhood asthma, of new-onset asthma in later life, and of asthma persisting into middle age [12].

The progression of asthma into adulthood maybe determined by different environmental and non-environmental exposures during early childhood [24]. The accommodation type does show a significant association between infant eczema and childhood and adult asthma. However, in homeowner’s type, the effect size is slightly higher compared to those who rent from council for unadjusted and adjusted models. The timing and extent of multiple environmental exposures may also result in different health outcomes [25]. This was evident at age 44 years, where smoking appeared to play a greater role in influencing asthma outcomes when measured by spirometry, although this may be biased by reverse causation.

The associations between infant eczema and asthma continued in our models that were stratified by sex. Men had an almost doubled risk of asthma (OR 4.0; 95% CI 3.5–4.3) compared to women (OR 2.1; 95% CI 1.8–2.4) for those who had infant eczema, which corresponds to previous findings (as described above). For example, in children, boys with eczema in the first 2 years of life had a 2.5-fold increased risk of having asthma, but there was no association between eczema and girls in this population [26]. In adulthood, women have a higher prevalence of eczema, but a lower prevalence of eczema with asthma or allergy [27]. It is possible that these differences may be a result of gender susceptibility to developing asthma, which results from a combination of gene and gender-specific differences in environmental exposures [28]. Housing type appeared to be a significant modifiable risk factor in our stratified models. This is important to consider because of the amount of time people spend indoors and because exposure to diverse indoor biological, chemical, and physical agents has been associated with allergic diseases [25].

### 4.3. Strengths and Weaknesses

The strengths of the study include the use of longitudinal data spanning 50 years, making it one of the longest running studies of its kind worldwide. Adjusting for age or season of birth was not necessary as all participants were recruited during the same week in 1958. An additional strength of this study was the use of GEE to overcome some of the limitations of logistic regression models and to account for correlations between repeated measurements for the same participant. The large size of this cohort allowed stratified analyses to examine whether the risk of asthma with infant eczema exposure was modified by sex or accommodation type. To avoid the potential of bias resulting from participants not fully completing the questionnaires, we eliminated all those missing data about their asthma symptoms across waves; moreover, asthma cases at age 16 years were removed from the analysis of the longitudinal data to avoid the duplicated asthma cases between ages 11 and 16 years due to questions possibly capturing cases twice.

Some limitations exist. The rate of participant dropout increased over time and it is not clear how this may have biased our results. The exclusion of participants who were lost to follow-up may introduce an element of bias as a result of people moving away and not responding. Previous work has suggested that the respondents in the biomedical survey at ages 44–45 years were representative of the surviving cohort sample, but not representative of the current UK population due to post-sampling immigration and the diversification of ethnic mix [29]; in the second wave in 1969 (first time recorded), the data dictionary lists participants as 97.8% Euro/Caucasian [30].

Also, as indicated above, the inclusion of self-reported outcome definitions resulted in higher effect estimates when compared to the spirometry results at age 44 years, which reduces our confidence in these results. Furthermore, the inclusion of self-reported outcomes may lead to prevalence estimates that include milder forms of these diseases when compared to doctor or spirometry diagnosis. This may also be further compounded by the use of different case definitions across the waves. Moreover, the reliance on children’s parents/guardians to complete the survey during childhood may further impact our findings due to recall bias (i.e., risk of memory decay as questions were retrospective).

Another limitation of this study is that there is no history of parental allergy/asthma, which is important to consider because having a family history of allergic diseases is a strong determinant of asthma [31], as at the commencement of the study (1958) there was little evidence for the genetic basis of asthma. The methods/accuracy in diagnosing asthma [32], use of “wheezing” as a case definition (an accepted definition in large-scale studies, cohort studies, or clinical trials) [33,34,35,36], and differences between parental and healthcare professional definitions of “wheezing” [29,30] limits our ability to understand the aetiology of this complex heterogeneous disease [37,38]. Results from European birth cohorts have suggested that to define asthma requires the presence of at least two of the following three criteria: (i) wheeze in last 12 months; (ii) asthma medication in last 12 months; and (iii) doctor-diagnosis of asthma (ever). However, for adults it may be more relevant to include shortness of breath or dry cough at night instead of wheeze [39].

This further highlights the need for the development of a more accurate case definition that is used consistently across future research and over time, and incorporates new thinking on asthma endotypes. Improvements in outcome measures are required to take place alongside the integration of molecular approaches and more complex longitudinal modelling to further our understanding of the underlying mechanism associated with the asthma transition from childhood to adulthood [40].

## 5. Conclusions

Eczema was one of the important factors influencing the risk of childhood and adult asthma in this population, using self-reported data and lung measures (spirometry). The effect size of infant eczema varied based on the outcome measure of asthma. Using spirometry data, the risk factor of infant eczema was smaller than that indicated by the self-reported data. This study highlights how differing measures of asthma may lead to different outcomes in asthma studies.

## Figures and Tables

**Table 1:** Summary statistics of covariates across asthma outcome(s) from age 11 to 50 years according to the participants’ responses to the birth cohort study survey.

	Current Asthma					
	Age 11 Years *n* (%; 95% CI)	Age 16 Years *n* (%; 95% CI))	Age 23 Years *n* (%; 95% CI)	Age 33 Years *n* (%; 95% CI)	Age 44 Years ** n (%; 95% CI)	Age 50 Years *n* (%; 95% CI)
**Eczema: *n* (percent)**	199 (1.7; 1.6–1.8)	147 (1.5; 1.4–1.6)	130 (1.2; 1.1–1.3)	211 (2.2; 2.1–2.3)	85 (1.0; 0.9–1.4)	91 (1.1; 1.0–1.2)
**Participant’s father’s occupation**						
**Group I ^1^**	232 (15.3; 15.2–15.5)	185 (15.6; 15.4–15.8)	551 (51.8; 51.2–51.4)	381 (14.6; 14.5–14.7)	164 (13.6; 13.4–13.8)	111 (13.0; 12.7–13.3)
**Group II ^2^**	768 (50.7; 50.6–50.8)	620 (52.1; 52.0–52.2)	213 (20.0; 19.7–20.3)	1398 (53.5; 53.4–53.6)	660 (55.0; 54.8–55.0)	454 (53.3; 53.4–53.6)
**Group III ^3^**	320 (21.1; 21.0–21.2)	238 (20.0; 19.8–20.2)	137 (12.9; 13.6–14.2)	522 (12.0; 11.9–12.1)	230 (19.1; 18.9–19.3)	176 (20.7; 20.5–20.9)
**Group IV^4^**	196 (12.9; 12.7–13.1)	147 (12.4; 12.2–12.6)		311 (11.9; 11.8–11.1)	149 (12.4; 12.2–12.6)	111 (13.0; 12.9–13.1)
**Accommodation type at age 7 years**						
**Owner occupied**	656 (53.4; 53.3–53.5)	514 (53.9; 53.8–54.0)	459 (53.0; 52.9–53.1)	1058 (51.1; 51.1–51.2)	517 (52.7; 52.6–52.8)	345 (50.3; 50.2–50.4)
**Council housing and privately rented**	573 (46.6; 46.5–46.7)	439 (46.1; 46.0–46.2)	407 (47.0; 46.9–47.1)	1012 (48.9; 48.8–49.9)	464 (47.3; 47.2–47.4)	341 (49.7; 49.6–49.8)
**Sex**						
**Male**	979 (58.9; 59.8–59.0)	799 (60.2; 60.1–60.3)	509 (43.1; 43.0–43.2)	1408 (48.7; 48.7–48.8)	672 (49.7; 49.6–49.8)	397 (41.7; 41.6–41.8)
**Female**	684 (41.1; 41.0–41.2)	528 (39.8; 39.7–39.9)	672 (56.9; 56.8–57.0)	1482 (51.3; 51.2:51.4)	680 (50.3; 50.2–50.4)	555 (58.3; 58.2–58.4)
**Mother smoked after month four of pregnancy?**						
**Abstain**	1043 (66.3; 66.1–66.4)	844 (68.0; 67.9–68.1)	728 (65.6; 65.5–65.7)	1739 (63.8; 63.7–63.9)	796 (63.4; 63.3–63.5)	552 (62.0; 61.9–62.1)
**Smoke**	530 (33.7; 33.6–33.8)	397 (32.0; 31.9–32.1)	381 (34.4; 34.3–34.5)	989 (36.3); 36.2–36.4)	460 (36.6; 36.5–36.7)	338 (38.0; 37.9–38.1)
**Mother’s weight**						
**Under 57 kg**	1103 (71.0; 70.9–71.1)	875 (71.3; 71.2–71.4)	788 (71.8;70.7–71.9)	1927 (71.4; 71.4–71.5)	904 (73.1; 73.0–73.1)	631 (71.7; 71.5–71.9)
**Between 57 kg and under 76 kg**	354 (22.8; 22.7–22.9)	283 (23.1; 23.0–23.2)	245 (22.3;22.1–22.5)	631 (23.4; 23.1–23.7)	257 (20.8; 20.6–21.0)	207 (23.5; 22.5–24.5)
**From 176 kg to above**	96 (6.2; 5.9–6.5)	70 (5.7; 5.4–6.0)	64 (5.8; 5.4–6.2)	141 (5.2; 5.1–5.2)	75 (6.1; 5.8–6.4)	42 (4.8; 4.7–4.8)
**No. of cigarettes parents smoked per week**						
**None**	799 (67.5; 6.7–6.8)	710 (65.3; 65.2–65.4)	534 (60.9; 60.7–60.9)	1,201 (55.4; 55.3–55.4)	620 (60.5; ;60.4–60.6)	439 (61.0; 60.7–61.3)
**≤29 a week**	185 (15.6; 15.4;15.8)	179 (16.5; 16.3–16.7)	176 (20.0; 19.8–20.2)	469 (21.6; 21.5–21.7)	195 (19.0; 18.8–19.2)	157 (21.8; 21.5–22.1)
**>29 a week**	199 (16.8; 16.6–17.0)	199 (18.3; 18.1–18.5)	169 (19.2; 19.0–19.4)	497 (22.9; 22.7–23.1)	210 (20.5; 20.3–20.7)	124 (17.2; 16.8–17.6)
**Birth weight (kg)**						
**≤2.5**	141 (8.5; 8.3–8.7)	113 (8.5; 8.3–8.7)	116 (9.8; 9.5–10.1)	266 (9.2; 9.1–9.3)	102 (7.5; 7.2–7.8)	95 (10.0; 9.0–10.0)
**> 2.5**	1522 (91.5; 91.4–91.6)	1214 (91.5; 91.4–91.6)	1065 (90.2; 90.1–90.3)	2624 (90.8; 90.7–90.9)	1250 (92.5; 92.4–92.5)	857 (90.0; 89.0–90.0)

^1^ Admin, professor, manager, higher admin, etc., ^2^ skilled workers, shopkeepers, clerical workers, personal workers, foremen; ^3^ semi-skilled workers, armed forces, shop assistants, farmers; and ^4^ unskilled workers.** The outcome(s) for diagnosis asthma were FEV1/FVC at age 44.

**Table 2:** Association between asthma and childhood eczema, adjusting for sex, socioeconomic status (SES), parental smoking, mother smoking status after month four of pregnancy, birth weight of subject, and mother’s weight at pregnancy period for ages 11, 16, 23, 33, and 50 years in the 1958BC study. The analysis was repeated after excluding the participants who smoked tobacco during the study.

Eczema	Model 1 (Unadjusted Model)		Model 4 (Adjusted Model)	
	All Data	Excluding Smokers	All Data	Excluding Smokers
	OR (95% CI)	OR (95% CI)	OR (95% CI)	OR (95% CI)
**Age 11 years**	3.76 (3.14–4.49)	-----	4.17 (3.30–5.29)	-----
**Age 16 years**	3.69 (3.00–4.52)	3.77 (3.03–4.69)	4.11 (3.20–5.28)	4.12 (3.15–5.38)
**Age 23 years**	3.12 (2.53–3.84)	3.47 (2.65–4.56)	3.49 (2.68–4.55)	4.08 (2.89–5.74)
**Age 33 years**	2.32 (1.92–2.79)	2.59 (2.10–3.21)	2.43 (1.91–3.09)	2.71 (2.06–3.56)
**Age 50 years**	2.55 (2.00–3.25)	2.83 (2.15–3.73)	3.06 (2.25–4.16)	3.07 (2.17–4.34)

Model 1 investigates the association between asthma and eczema; Model 4 (adjusted model) is adjusted for sex, SES (father’s occupation and accommodation at age 7 years), parental smoking status, mother smoking during pregnancy after month four, child weight at birth, and mother’s weight. Note that to identify the questionnaire questions for asthma at ages 11, 16, 23, 33, and 50 years, see Figure 1. The number of excluded smokers were 217 (16.5%), 579 (65.7%), 959 (46.8%), 492 (37.8%), and 273 (28.8%) subjects at ages 16, 23, 33, 44, and 50 years, respectively.

**Table 3:** Association between asthma and childhood eczema adjusting for sex, SES, parental smoking, mother smoking status after month four of pregnancy, birth weight of subject, and mother’s weight at pregnancy period for ages 44–46 years for biochemical data. The analysis was repeated after excluding the participants who smoked tobacco during the study.

Eczema	Model 1 (Unadjusted Model)		Model 4 (Adjusted Model)	
	All Data	Excluding Smokers	All Data	Excluding Smokers
	OR (95% CI)	OR (95% CI)	OR (95% CI)	OR (95% CI)
Age 44 years * Spirometry data	1.36 (1.06–1.75)	1.69 (1.27–2.25)	1.29 (0.91–1.79)	1.61 (1.09–2.32)

Model 1 investigates the association between asthma and eczema; Model 4 (adjusted model) is adjusted for sex, SES (father’s occupation and accommodation at age 7 years), parental smoking status, mother smoking during pregnancy after month four, child weight at birth, and mother’s weight. * The outcome(s) for diagnosis asthma were FEV1/FVC at age 44.

**Table 4:** Follow-up data of individuals with asthma/wheezing or bronchitis according to age of onset.

New Cases Examined at	Number of Cases in Whom Asthma Was Still Present (%)			
	Age 16 Years	Age 23 Years	Age 33 Years	Age 50 Years
Age 11 years	701/1327 (53%)	367/1181 (31%)	603/2890 (21%)	229/952 (24%)
Age 16 years	-----	364/1181 (31%)	562/2890 (20%)	206/952 (22%)
Age 23 years	-----	-----	651/2890 (23%)	307/952 (32%)
Age 33 years	-----	-----	-----	532/952 (56%)
Age 50 years	-----	-----	-----	-----

**Table 5:** Odds ratio (95% CI) for the association between infant eczema and onset of asthma later in childhood and as adults by using a repeated measures multilevel model for the longitudinal analysis to account for the nesting of repeated asthma events within individuals, and potential variation between waves or ages (results stratified by sex, accommodation type).

Eczema	Model 1 (Unadjusted Model)		Model 4 (Adjusted Model)	
	All Data	Excluding Smokers	All Data	Excluding Smokers
	OR (95% CI)	OR (95% CI)	OR (95% CI)	OR (95% CI)
All data *	2.91 (2.64, 3.22)	3.18 (2.87, 3.48)	3.19 (2.80, 3.62)	3.43 (3.01, 3.99)
**Sex:**				
**Male**	4.04 (3.52,4.36)	4.29 (3.72,4.83)	3.97 (3.31,4.75)	4.12 (3.65,4.99)
**Female**	2.05 (1.77,2.39)	2.23 (1.99,2.51)	2.58 (2.13,3.12)	2.78 (2.38,3.42)
**Accommodation type**				
**Owner occupied**	3.25 (2.84,3.73)	3.45 (3.04, 3.99)	3.39 (2.88,3.98)	3.56 (3.01,4.02)
**Council rented**	2.56 (2.14, 3.06)	2.89 (2.41, 3.49)	2.84 (2.28, 3.53)	3.28 (2.63, 3.99)

Model 1 investigates the association between asthma and eczema; Model 4 (adjusted model) is adjusted for sex, SES (father’s occupation and accommodation at age 7 years), parental smoking status, mother smoking during pregnancy after month four, child weight at birth, and mother’s weight. Note that to identify the questionnaire questions for asthma at ages 11, 16, 23, 33, and 50 years, see Figure 1. The number of excluded smokers were 579 (65.6%), 959(46.9%), 492 (37.8%), and 273 (28.8%) subjects at ages 23, 33, 44, and 50 years, respectively. * All data refers to the pooling all of waves or ages together, which fitted the models without classification. Note that asthma cases at age 16 years were removed to avoid the duplicated data between ages 11 and 16 years, since the questionnaire question was “Have you ever have asthma or wheezing?”.
